# Exploration and validation of hub genes and pathways in the progression of hypoplastic left heart syndrome via weighted gene co-expression network analysis

**DOI:** 10.1186/s12872-021-02108-0

**Published:** 2021-06-15

**Authors:** Xuelan Liu, Honglei Shang, Bin Li, Liyun Zhao, Ying Hua, Kaiyuan Wu, Manman Hu, Taibing Fan

**Affiliations:** 1 Department of Children’s Heart Center, Henan Provincial People’s Hospital, Department of Children’s Heart Center of Fuwai Central China Cardiovascular Hospital, Central China Fuwai Hospital of Zhengzhou University, Zhengzhou, 450003 Henan China; 2 Department of Radiology, The Third Affiliated Hospital of Zhengzhou University, Zhengzhou, 450052 China

**Keywords:** Hypoplastic left heart syndrome, Weighted gene co-expression network analysis, Protein–protein interaction network, Hub genes, Pathways

## Abstract

**Background:**

Despite significant progress in surgical treatment of hypoplastic left heart syndrome (HLHS), its mortality and morbidity are still high. Little is known about the molecular abnormalities of the syndrome. In this study, we aimed to probe into hub genes and key pathways in the progression of the syndrome.

**Methods:**

Differentially expressed genes (DEGs) were identified in left ventricle (LV) or right ventricle (RV) tissues between HLHS and controls using the GSE77798 dataset. Then, weighted gene co-expression network analysis (WGCNA) was performed and key modules were constructed for HLHS. Based on the genes in the key modules, protein–protein interaction networks were conducted, and hub genes and key pathways were screened. Finally, the GSE23959 dataset was used to validate hub genes between HLHS and controls.

**Results:**

We identified 88 and 41 DEGs in LV and RV tissues between HLHS and controls, respectively. DEGs in LV tissues of HLHS were distinctly involved in heart development, apoptotic signaling pathway and ECM receptor interaction. DEGs in RV tissues of HLHS were mainly enriched in BMP signaling pathway, regulation of cell development and regulation of blood pressure. A total of 16 co-expression network were constructed. Among them, black module (r = 0.79 and *p* value = 2e−04) and pink module (r = 0.84 and *p* value = 4e−05) had the most significant correlation with HLHS, indicating that the two modules could be the most relevant for HLHS progression. We identified five hub genes in the black module (including *Fbn1*, *Itga8*, *Itga11*, *Itgb5* and *Thbs2*), and five hub genes (including *Cblb*, *Ccl2*, *Edn1*, *Itgb3* and *Map2k1*) in the pink module for HLHS. Their abnormal expression was verified in the GSE23959 dataset.

**Conclusions:**

Our findings revealed hub genes and key pathways for HLHS through WGCNA, which could play key roles in the molecular mechanism of HLHS.

## Introduction

Hypoplastic left heart syndrome (HLHS) is a group of complex congenital heart malformations characterized by severe stenosis or atresia of the aortic valve and mitral valve, ascending aorta, and left ventricular dysplasia [7, 12, 19]. It was first proposed by Noonan and Nadas [29]. HLHS accounts for 1.4–4.1% of congenital cardiovascular malformations, and the prevalence of live births is about 2:10,000–3:10,000 [16]. The dysplastic left ventricle cannot provide adequate systemic circulation perfusion and oxygen supply. Oxygenated blood bypasses the dysplastic left heart to supply the whole body through an atrial septal defect and an open arterial catheter, followed by symptoms such as strenuous breastfeeding, difficulty breathing, rapid heartbeat, pulse weakness, dull skin or cyanosis, and severe heart failure. HLHS accounts for 25% of all babies who die of congenital heart diseases. Without diagnosis and treatment, 95% of babies die within one month after birth [10]. The treatment of HLHS remains extremely challenging. Patients usually undertake aggressive palliative operations. Despite the diagnosis and treatment of the disease have made great progress since the first implementation of Norwood surgery in 1983, the morbidity and mortality of HLHS patients are still high [23].

The exact cause of HLHS is still unclear. Increasing evidence suggests that genetic pathology is involved in the progression of HLHS. Approximately 30% children with HLHS have genetic syndrome or other extracardiac abnormalities [27]. Various syndromes caused by chromosomal abnormalities are also associated with HLHS, including Turner syndrome (X chromosome monomer), Edwards syndrome (Trisomy 18) and DiGeorge syndrome (22q11.21 deletion) [40]. It has been reported that several specific genes are related to HLHS such as *HAND1*,* TBX5*, *FOXC2*, *GJA1*, *NKX2-5*, *NOTCH1*, *MYH6* and* ERBB4* [6]. Theis et al. demonstrated that the compound heterozygous mutation of the allele *NOTCH1* is the basis of impaired cardiac development in patients with HLHS [37]. During human embryonic development, *TAB2* is abundantly expressed in the endocardial pad, which plays an important role in the outflow tract and valve formation. *TAB2* haploinsufficiency is also a risk factor for HLHS [6]. Therefore, genomics analysis is of great significance for the precise management and treatment of HLHS.

Weighted correlation network analysis (WGCNA), as a systematic biological method, may describe the pattern of gene association between different samples [28]. It can be used to identify highly synergistically changing gene sets, and to identify potential biomarkers and therapeutic targets based on the interconnectivity of gene sets and the association between gene sets and phenotypes. Compared with only focusing on DEGs, WGCNA utilizes the information of thousands of genes with the most changes or all genes to identify gene sets of interest and to perform correlation analysis with phenotypes [5]. The advantages of WGCNA are as follows: one is to make full use of information, and the other is to convert the association of thousands of genes and phenotypes into the association of several gene sets and phenotypes, eliminating the problem of multiple hypothesis test correction [39]. In this study, we firstly constructed a co-expression network for HLHS. Furthermore, we identified hub genes and pathways for HLHS progression, which deserve further research in more basic experiments and clinical research.

## Materials and methods

### Data acquisition and preprocessing

**Fig. 1:**
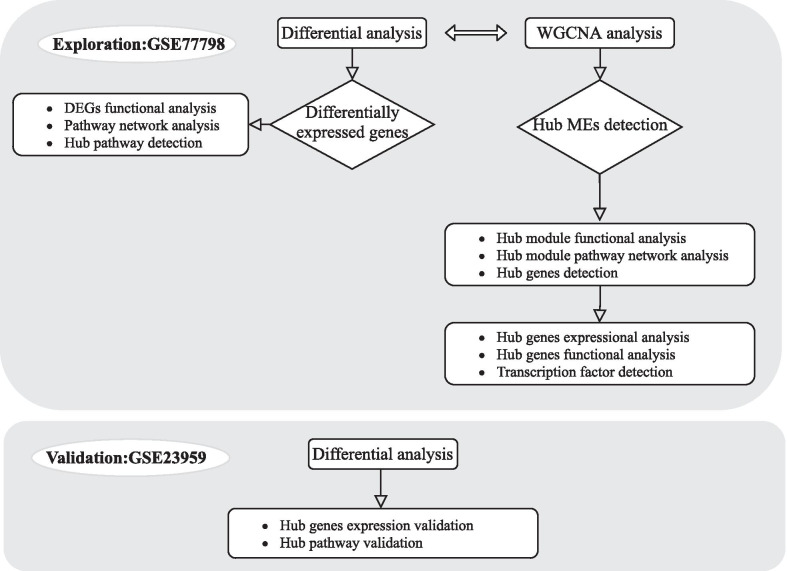
The workflow of this study: data acquisition, preprocessing, analysis and verification

The workflow of this study is shown in Fig. 1. Two HLHS mRNA expression profiling datasets were downloaded from the Gene Expression Omnibus (GEO; https://www.ncbi.nlm.nih.gov/geo). GSE77798 RNA-seq expression profiling dataset was composed of 16 left ventricle (LV) and right ventricle (RV) from HLHS mice (n = 6) and littermate controls (n = 10). The dataset was based on GPL13112 Illumina HiSeq 2000 (*Mus musculus*) [22]. GSE23959 microarray expression profiling dataset included 6 RV samples from HLHS neonates and 10 LV and RV from controls on the GPL5188 [HuEx-1_0-st] Affymetrix Human Exon 1.0 ST Array [probe set (exon) version] platform. Quality control was achieved via arrayQualityMetrics package in R [18]. Robust multi-array average (RMA) background correction was performed on the raw expression data. The processed signals were transformed into log2, followed by quantile normalization. The robust K-Nearest Neighbor (KNN) algorithm was executed to process missing values [14].

### Differential expression analysis

Differentially expressed genes (DEGs) were screened between HLHS and control samples using Linear Models for Microarray Data (limma) package in R [33]. False discovery rate (FDR) was calculated via Benjamini–Hochberg method. log2|fold change (FC)|> 1 and FDR < 0.05 were set as the threshold value.

### Functional enrichment analysis

Gene Ontology (GO) and Kyoto Encyclopedia of Genes and Genomes (KEGG) were analyzed via Metascape online database (http://metascape.org/gp/index.html) [42]. Metascape integrates more than 40 bioinformatics knowledge bases into a single user interface, which provides comprehensive gene list annotation and analysis resources.

### WGCNA

WGCNA package in R was utilized to construct co-expression network [20]. The gene co-expression similarity matrix was composed of the absolute values of the correlation coefficients between genes. For continuous variables of genes, Pearson correlation coefficients were used (the range of correlation coefficients is [0, 1]), and the correlation matrix was as follows: S = [S_i,j_] (i, j refers to the ith, jth gene). Soft threshold value was calculated through an exponential adjacency function, as follows: a_i,j_ = power (S_i, j_, β) =|S_i, j_|^β^ (a_i,j_ is the adjacency function between the ith and jth genes; β refers to soft threshold value). To make the co-expression network in line with the characteristics of scale-free network, soft threshold value was screened under the threshold of R^2^ > 0.8. Then, correlation matrix S = [S_i,j_] was converted into adjacency matrix A = [A_i,j_] by pickSoftThreshold function. The topological overlap dissimilarity measure (TOM) was used to calculate the degree of correlation between genes, as follows: $TOM_{{IJ}} = \frac{{\mathop \sum \nolimits_{u} a_{{iu}} a_{{uj}} + a_{{ij}} }}{{\min \left( {ki,~kj} \right) + 1 - a_{{ij}} }}$ ($a_{{ij}}$ is [0, 1]). Gene modules were assigned based on the degree of connection between modules. Therefore, TOM was converted into dissimilarity degree, as follows: dissTOM_ij_ = 1 − TOM_ij_. Then, a hierarchical clustering tree diagram of genes was constructed and gene modules were assigned using dynamic cutting method. Afterwards, we calculated eigengene that refers to the first principal component of all gene expression level vectors in the modules. Correlation between eigengene of each module and clinical traits including HLHS and heart region was analyzed.

### PPI network

Interested genes were imported into STRING online database (version 11; http://string-db.org/) [35]. Protein–protein interactions were visualized using Cytoscape (version 3.8.0) [9].

### External dataset validation

The expression patterns of hub genes between HLHS and controls were validated using an external GSE23959 microarray expression profiling dataset.

## Results

### Identification of DEGs and pathways for LV and RV HLHS

**Fig. 2:**
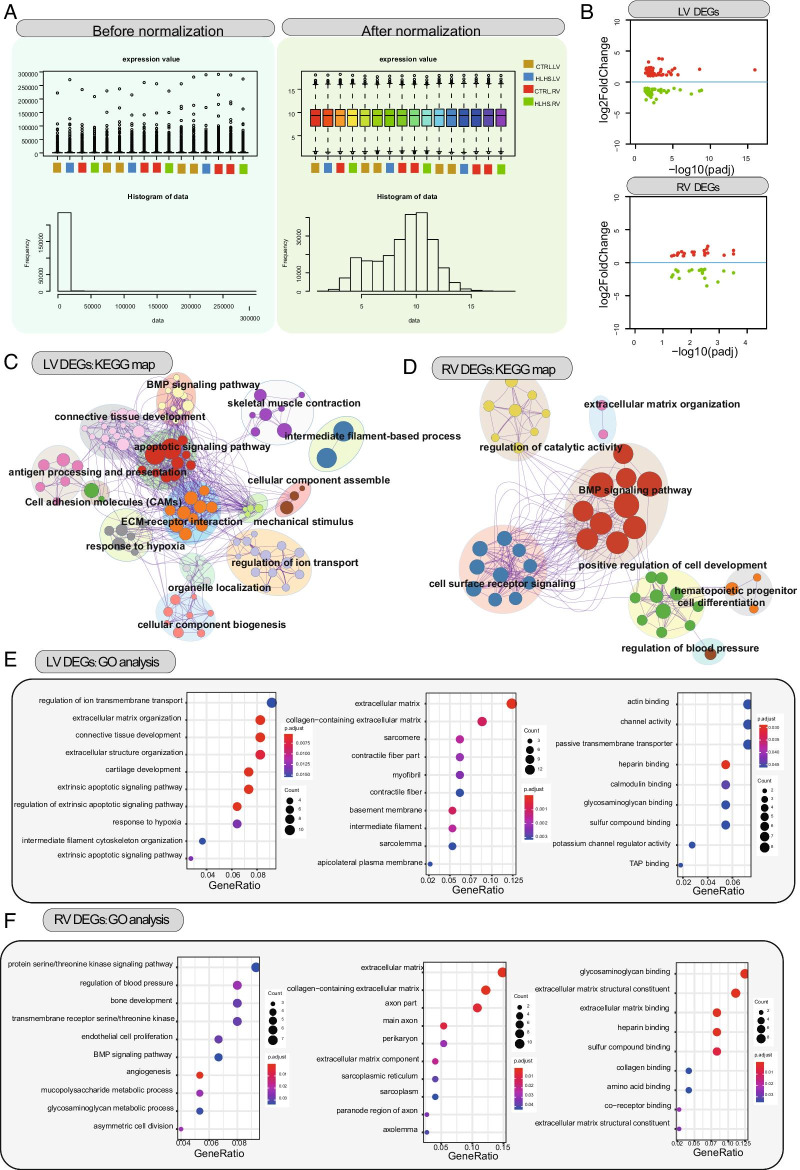
Identification of DEGs and pathways for LV and RV HLHS. **A** Data normalization. Before normalization (left), after normalization (right) and histogram of data (bottom). **B** DEGs between LV/RV and controls. Red represents up-regulation and green represents down-regulation. **C** KEGG pathway enrichment map of DEGs in LV HLHS. **D** KEGG pathway enrichment map of DEGs in RV HLHS. **E** GO enrichment analysis results of DEGs in LV HLHS. **F** GO enrichment analysis results of DEGs in RV HLHS. The size of the circle is proportional to the number of genes enriched. The shade of the circle color represents adjusted *p* value

To eliminate the changes in intensity caused by the experimental technique, and to make the data of each sample and parallel experiment at the same level, GSE77798 RNA-seq expression profiling data were pre-processed, including data filtering, normalization, logarithmization, and estimation of missing values. GSE77798 RNA-seq expression profiling was processed by quantile normalization (Fig. 2A). Following normalization, under the threshold of FDR < 0.05 and log2|FC|> 1, we identified 44 up-regulated and 44 down-regulated genes between LV HLHS and controls (Fig. 2B). Furthermore, 21 up-regulated and 20 down-regulated genes were screened between RV HLHS and controls. KEGG enrichment analysis results showed that DEGs in LV HLHS were distinctly enriched in several key pathways including connective tissue development, BMP signaling pathway, skeletal muscle contraction, intermediate filament-based process, apoptotic signaling pathway, extracellular matrix (ECM)-receptor interaction, cellular component assemble, mechanical stimulus, regulation of ion transport, organelle localization, response to hypoxia, antigen processing and presentation and cell adhesion molecules (CAMs) in Fig. 2C. Moreover, DEGs in RV HLHS were significantly involved in regulation of catalytic activity, extracellular matrix organization, BMP signaling pathway, positive regulation of cell development, hematopoietic progenitor cell differentiation, regulation of blood pressure and cell surface receptor signaling (Fig. 2D). GO enrichment analysis including biological process (BP), cellular component (CC) and molecular component (MF) was performed. As shown in Fig. 2E, DEGs in LV HLHS were most significantly enriched in regulation of ion transmembrane transport, extracellular matrix and actin binding. Furthermore, DEGs in RV HLHS were most significantly enriched in protein serine/threonine kinase signaling pathway, extracellular matrix and glycosaminoglycan binding (Fig. 2F).

### Construction of a co-expression network and key modules for HLHS

**Fig. 3:**
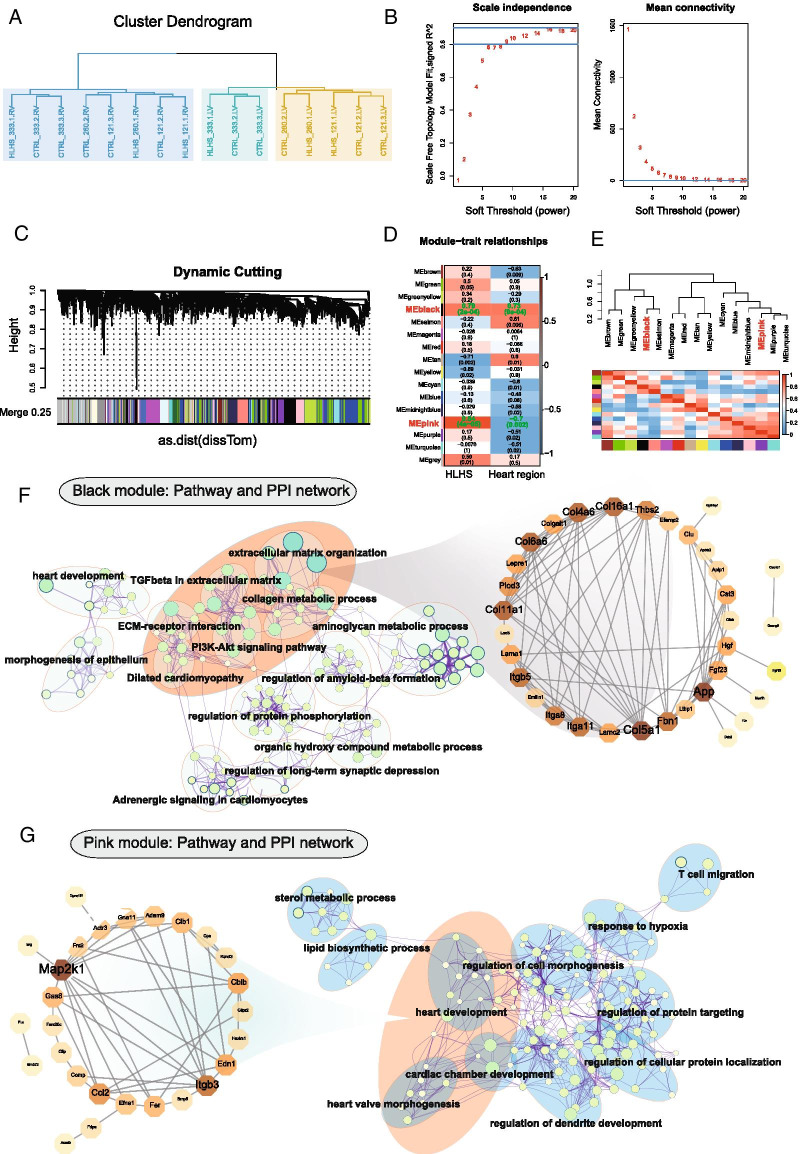
Construction of a co-expression network and key modules for HLHS. **A** Cluster dendrogram. **B** Analysis of the scale-free topology model fit index and mean connectivity for different soft threshold values. The horizontal axis is the soft threshold (power), and the vertical axis is the evaluation parameter of the scale-free network (left) or mean connectivity (right). **C** Gene dendrogram via average linkage hierarchical clustering. Different colors exhibit the module assignment through the dynamic tree cutting. **D** Heat maps showing the module-trait relationships. Each cell contains correlation coefficient and *p* value. Red indicates positive correlation and blue indicates negative correlation. **E** Eigengene adjacency heatmap. Each row and each column respectively correspond to one module eigengene. Green indicates low adjacency and red indicates high adjacency. The diagonal and red rectangle represents one meta-module. **F**, **G** KEGG pathway enrichment map and PPI network for the black and pink modules. For PPI network, the color shade of nodes is proportional to degree

As shown in Fig. 3A, there were no outlier samples. To ensure a scare free network, soft threshold value (β) was set as 8 (R^2^ = 0.8) in Fig. 3B. DEGs with similar expression patterns were assigned into modules by average link clustering and dynamic tree cutting methods (Fig. 3C). Finally, 16 co-expression modules were conducted for HLHS. The correlation results among the gene profile and the sample trait of HLHS and heart region were presented in Fig. 3D. The black module and pink module had the most significant correlation with HLHS (MEblack: r = 0.79 and *p* value = 2e−04; MEpink: r = 0.84 and *p* value = 4e−05) and heart region (MEblack: r = 0.73 and *p* value = 9e−04; MEpink: r = −0.70 and *p* value = 0.002) basing on the gene significance (GS) algorithm. Thus, the two modules were considered the most relevant for HLHS progression. There were 249 genes in the black module and 222 genes in the pink module. Figure 3E shows the relationships among different modules through an eigengene adjacency heatmap. To further validate the roles of the two modules in HLHS progression, we carried out KEGG pathway enrichment and PPI network analyses. In Fig. 3F, the genes in the black module were most significantly enriched in TGFβ in extracellular matrix, collagen metabolic process, ECM receptor interaction, PI3K-Akt pathway and dilated cardiomyopathy, especially extracellular matrix organization. The genes in the extracellular matrix organization pathways were used to conduct a PPI network. We found that *Fbn1* (degree = 13), *Thbs2* (degree = 11), *Itga8* (degree = 9), *Itga11* (degree = 9) and *Itgb5* (degree = 9) could be hub genes for HLHS. For the genes in the pink module, heart development, heart valve morphogenesis and cardiac chamber development were the most significantly enriched pathways (Fig. 3G). Among the genes in the three pathways, five hub genes were identified including *Map2k1* (degree = 8), *Ccl2* (degree = 7), *Itgb3* (degree = 5), *Edn1* (degree = 4) and *Cblb* (degree = 4) through PPI network.

### Identification and validation of hub genes for HLHS

**Fig. 4:**
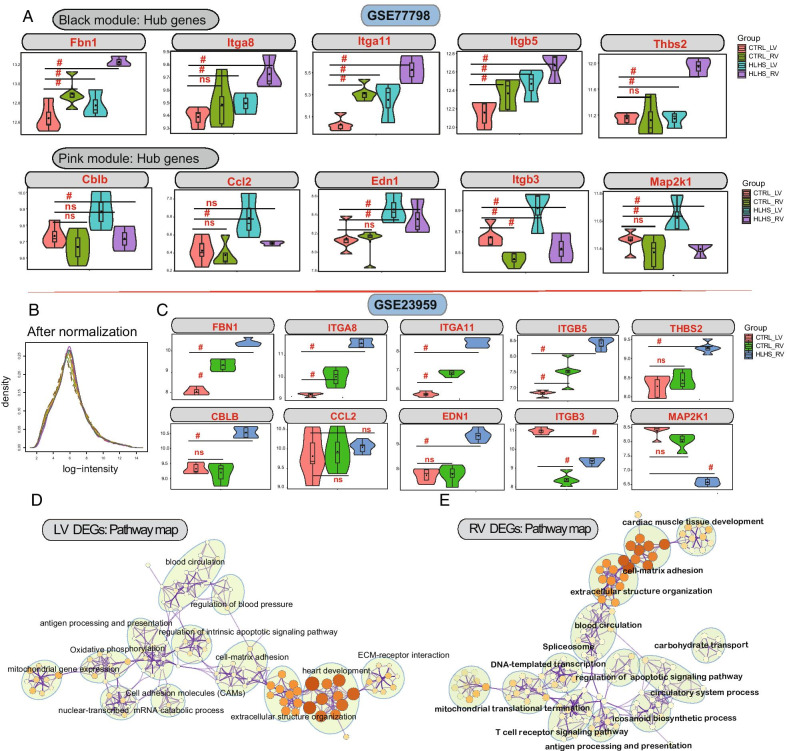
Identification and validation of hub genes for HLHS. **A** The expression patterns of hub genes from the black and pink modules between LV/RV HLHS and controls in the GSE77798 dataset. **B** Data normalization from the GSE23959 dataset. **C** The expression patterns of hub genes from the black and pink modules between LV/RV HLHS and LV controls in the GSE23959 dataset. #*p* value < 0.05; *ns* no statistical significance. **D**, **E** KEGG pathway enrichment analysis of LV and RV DEGs in the GSE23959 dataset

We examined the expression patterns of these hub genes in the black and pink modules from PPI networks between HLHS and controls in the GSE77798 dataset. For hub genes in the black module, compared to LV controls, the expression of *Fbn1*, *Itga11* and *Itgb5* were significantly higher in RV controls, LV HLHS and RV HLHS (all *p* values < 0.05) in the GSE77798 dataset (Fig. 4A). Furthermore, Itga8 and Thbs2 had higher expression levels in LV HLHS and RV HLHS than in LV controls (all *p* values < 0.05). For hub genes in the pink module, compared to LV controls, the expression *Cblb* was distinctly higher in RV HLHS (*p* value < 0.05). *Ccl2* had a significantly higher expression in LV HLHS compared to LV control (*p* value < 0.05). In comparison to LV controls, the expression of *Itgb3* was significantly higher in LV HLHS (*p* value < 0.05). However, its expression was distinctly lower in RV controls and RV HLHS compared to LV controls (both *p* value < 0.05). *Edn1* and *Map2k1* exhibited higher expression levels in LV and RV HLHS than in LV controls (all *p* values < 0.05).

After normalization (Fig. 4B), the expression patterns of these hub genes were further validated in the GSE23959 dataset. Compared to LV controls, the expression of *Fbn1*, *Itga8*, *Itga11* and *Itgb5* had higher expression levels in RV controls and RV HLHS (all *p* values < 0.05) in Fig. 4C. Furthermore, *Thbs2* expression was significantly higher in RV HLHS in comparison to LV controls (*p* value < 0.05). Compared with LV controls, the expression of *Cblb* and *Edn1* in RV HLHS (both *p* values < 0.05). *Itgb3* had lower expression levels both in RV controls and RV HLHS in comparison to LV controls (all *p* values < 0.05). Moreover, *Map2k1* had a lower expression level in RV HLHS than in LV controls (*p* value < 0.05). KEGG pathway enrichment analysis of all DEGs was presented using the GSE23959 dataset. As shown in Fig. 4D, LV DEGs were most significantly enriched in the heart development and extracellular structure organization. Additionally, cell–matrix adhesion and extracellular structure organization were the most significant pathways for RV DEGs (Fig. 4E).

## Discussion

HLHS is characterized by LV hypoplasia and increased biomechanical pressure on RV by single ventricular physiology [32]. By analyzing HLHS gene RNA-seq profile data, we obtained 14,889 genes for HLHS. The expression profiles of these genes were used as data sources to perform WGCNA on HLHS and a total of 16 co-expressed gene modules were identified in this study. Among them, black module and pink module were the two modules most relevant to HLHS. Based on the genes in these two modules, we constructed PPI networks. Finally, 10 hub genes were confirmed for HLHS, which were differentially expressed in LV and RV tissues between controls and HLHS. By verifying these hub genes in the HLHS expression profile data of an independent cohort, we found that most of the genes were consistent in the HLHS cohort of different data sources, indicating that our analysis method was accurate and reproducible.

We identified five hub genes for HLHS in the black module, including *Fbn1*, *Itga8*, *Itga11*, *Itgb5* and *Thbs2*. *Fbn1* (Fibrillin-1) has been found to be associated with heart development [26]. Its mutation could increase genetic susceptibility to thoracic aortic aneurysms [15]. Furthermore, its mutation leads to Marfan syndrome (MFS) that is the most common hereditary connective tissue disease [31]. In this study, *Fbn1* had distinctly higher expression in LV/RV HLHS compared to controls. Furthermore, a significant difference in Fbn1 expression was detected between LV and RV for control mice and neonates. *Itga8* (Integrin Subunit Alpha 8) inhibits NFκB and JAK-STAT signaling and cardiac injury in myocardium without stress [3]. *Itga8* expression was significantly higher in LV/RV HLHS in comparison to controls both in the GSE77798 and GSE23959 datasets. *Itga11* (Integrin Subunit Alpha-11) expression has been detected to be increased in methylglyoxal-induced collagen-treated human cardiac fibroblasts and streptozotocin-treated Sprague–Dawley rat cardiac fibroblasts, which may promote the formation of pre-fibrotic fibroblasts and fibrotic stroma in diabetic cardiomyopathy [36]. *Itgb5* (Integrin Subunit Beta 5) has been identified to be in significant correlation with coronary artery disease and age-dependent organ fibrosis [4, 38]. *Thbs2* (Thrombospondin 2) mediates cell–matrix interactions, vascular integrity and thrombosis [13]. In our study, its expression was lower in LV HLHS than controls, which was higher in RV HLHS compared ton controls.

Five hub genes including *Cblb*, *Ccl2*, *Edn1*, *Itgb3* and *Map2k1* were screened for HLHS in the pink module. *Cblb* (Casitas B-cell lymphoma-B) is lowly expressed in plaques for human atherosclerosis, thereby leading to CD8^+^ T cell-induced macrophage death and accelerating atherosclerosis [34]. Our findings revealed that *Cblb* expression was significantly down-regulated in RV HLHS not LV HLHS in comparison to controls. It has been reported that targeting *Ccl2* (C–C Motif Chemokine Ligand 2) could ameliorate atherosclerosis [41]. Moreover, Dectin-2-mediated *Ccl2* in resident tissue macrophages can facilitate cardiac arteritis [25]. In mice, *Ccl2* expression was up-regulated in LV HLHS than controls. However, no significant difference in *Ccl2* expression was detected between HLHS and controls. *Edn1* (Endothelin 1) genetic locus is correlated to spontaneous coronary artery dissection [1]. It was highly expressed in LV/RV HLHS compared to controls in mice, and was highly expressed in RV HLHS than control neonates. *Itgb3* (Integrin Subunit Beta 3) is related to myocardial infarction risk [21]. *Map2k1* (Mitogen-Activated Protein Kinase Kinase 1) mutation is often in association with the clinical phenotype of the cardiovascular system skin syndrome [30]. Our study found that *Map2k1* was highly expressed in LV HLHS and lowly expressed in RV HLHS compared to controls in mice. Furthermore, its low expression was found in human RV HLHS in comparison to controls.

Complex life phenomena are the result of the interaction of a large number of biological components. Biological research has shifted from collecting gene and protein information to systematically using this information to clarify the synergy between them. In this study, we tried to probe into the molecular mechanism of HLHS through functional enrichment analysis of DEGs-related HLHS. DEGs in LV HLHS were distinctly involved in heart development, apoptotic signaling pathway and ECM receptor interaction. Abnormally expressed genes related to heart development could contribute to the progression of HLHS. Furthermore, imbalance of apoptotic signaling pathway in cardiomyocytes may be an important factor of HLHS. As a previous study, RV tissues in HLHS exhibit immature ECM and increased cardiomyocyte apoptosis [8]. Thus, the roles of these DEGs in HLHS need further exploration. DEGs in RV HLHS were mainly enriched in BMP signaling pathway, regulation of cell development and regulation of blood pressure. Dysregulation of the BMP pathway is the basis of many diseases of different organ systems in humans [24]. As a previous study, changes in gene expression in the BMP pathway has been found in RV tissues for neonates with HLHS [32]. Cardiomyocytes from neonates with HLHS exhibit multiple expression and function differences [17], which could be mediated by a variety of DEGs at a transcriptional level [11]. Our study found that DEGs in RV HLHS were involved in the regulation of blood pressure, as previous studies [2].

Facing the increasing amount of high-throughput data, it is a difficult problem about how to effectively extract useful information to obtain the regulatory relationship between genes in the research of systems biology. The regulatory relationship between genes has spatiotemporal specificity. In different organs, different physiological conditions and pathological states, and at different time points, this regulatory relationship will change accordingly. It is these changes that determine cell proliferation, differentiation, as well as occurrence, development of HLHS. The modularity of the biological network is the result of living organisms to achieve specific biological functions. Modularity provides us with a simple and effective method to understand the regulatory relationship between genes, which is an indispensable method in the research of systems biology. This study is the first to analyze HLHS data by WGCNA. Our results showed that WGCNA can discover biologically meaningful gene modules, and the hub genes related to the clinical information found are consistent with literature reports, which also proves the accuracy and effectiveness of WGCNA of gene expression data. Further excavation of information on gene modules will help us better understand the role and significance of hub genes, key signaling pathways, as well as the regulatory mechanisms between genes on the development of HLHS.

## Conclusion

In this study, we identified hub genes and key pathways for HLHS via WGCNA. These findings showed that these hub genes could play an important role in HLHS and cardiovascular diseases, which provided important clues for further revealing the molecular mechanism of HLHS.

## Data Availability

The Raw microarray datasets of GSE77798 and GSE23959 were downloaded from GEO database (http://www.ncbi.nlm.nih.gov/geo/). All of the analysis script are available with reasonable request from the corresponding author.

## Abbreviations

HLHS: Hypoplastic left heart syndrome
DEGs: Differentially expressed genes;
LV: Left ventricle
RV: Right ventricle
WGCNA: Weighted gene co-expression network analysis
PPI: Protein–protein interaction
GEO: Gene Expression Omnibus
FDR: False discovery rate
FC: Fold change
GO: Gene Ontology
KEGG: Kyoto Encyclopedia of Genes and Genomes
TOM: Topological overlap dissimilarity measure
BP: Biological process
CC: Cellular component
MF: Molecular component
